# Interferon gamma (IFNg) drives disease in the TLR9-mediated cytokine storm syndrome in mice

**DOI:** 10.1186/1546-0096-13-S1-O63

**Published:** 2015-09-28

**Authors:** C de Min, V Buatois, L Chatel, L Cons, F De Benedetti, M Kosco-Vilbois, W Ferlin

**Affiliations:** 1 NovImmune S.A., Plan-Les-Ouates, Switzerland; 2 Ospedale Bambino Gesù, Rome, Italy

## 

The Cytokine Storm Syndrome (CSS) is characterized by an overwhelming activation of immune cells observed in life-threatening disorders such as familial hemophagocytic lymphohistiocytosis (fHLH) and secondary (s) HLH/macrophage activation syndrome (MAS) as well as during serious infection. However, it is not known if the CSS can be attributed to a single cytokine. Increased blood levels of interferon gamma (IFNg) in HLH and sHLH/MAS patients potentially indicate a central role for this cytokine in the CSS. Using a mouse model that mimics an infection-driven CSS (i.e., CpG-ODN), our study showed that total IFNg levels originating within organs are 500 to 2,000-fold higher than those measured in peripheral blood as CSS develops. Ablation of IFNg activity in tissues led to the amelioration of the plethora of associated CSS clinical and laboratory parameters. Furthermore, the IFNg signature gene products, CXCL9 and CXCL10, correlated with disease severity in the mouse model of CSS and patients with sHLH. Thus, anti-IFNg targeted therapy should control diseases associated with the cytokine storm and we propose the use of CXCL9 or CXCL10 as a means to monitor total IFNg activity in patients.

